# The Effect of Past Cataract Surgery within the Medium to Long-Term Period on Patients with Dry Eye Disease

**DOI:** 10.3390/jcm11040972

**Published:** 2022-02-13

**Authors:** Lei Siew, Louis Tong

**Affiliations:** 1 Ocular Surface Research Group, Singapore Eye Research Institute, The Academia, 20 College Road, Singapore 169856, Singapore; siewlei_@u.nus.edu; 2 Yong Loo Lin School of Medicine, National University of Singapore, Singapore 117585, Singapore; 3 Eye-Academic Clinical Program, Duke-NUS Graduate Medical School, 8 College Road, Singapore 169857, Singapore; 4 Corneal and External Eye Disease, Singapore National Eye Centre, 11 Third Hospital Ave., Singapore 168751, Singapore

**Keywords:** cataract surgery, tear film, dry eye disease, dry eye symptoms, medium to long term, patient satisfaction

## Abstract

This study investigates the effects of cataract surgery on dry eye parameters of patients with dry eye disease (DED) in the medium- to long-term post-surgical period (6 months to 5 years). A cross-sectional study was conducted on 438 eyes on first visit to a tertiary clinic (219 eyes with cataract surgery within timeframe, i.e., pseudophakic dry eye group, 219 comparison eyes without such history, i.e., comparison dry eye group). Parameters evaluated include Ocular Surface Disease Index (OSDI) and standard examination for DED. A significantly greater proportion of pseudophakic dry eye group (50%) experienced frequent blurred vision (≥1 episode per week) compared to dry eye control group (38%) (OR = 1.66, 95%CI 1.13, 2.44). Those with ocular discomfort before surgery were more likely to experience blurring at least once a day. However, a significantly greater proportion of the pseudophakic dry eye group (34%) had Schirmer’s I >8mm compared to the comparison group (25%) (OR = 0.605, 95% CI 0.398, 0.921), though the difference was not clinically significant (7.51 mm versus 6.51 mm, *p* > 0.05). Other DED signs (e.g., overall OSDI score, Tear Break-up Time) were not found to be worse among pseudophakic dry eye group. Pre-operative counselling and preventative measures should be undertaken, especially those with suggestive symptoms pre-operatively.

## 1. Introduction

Cataract surgery is the most performed surgery in ophthalmology. The Singapore Epidemiology of Eye Disease Study showed that that 1 in 10 Singaporeans aged 40 years and above required cataract surgery in at least one eye over a period of 6 years [1]. With the development of new surgical methods and the use of smaller incisions, cataract surgery provides excellent clinical outcomes and is associated with shorter recovery periods [2]. This has led to higher patient expectations in visual improvement and quality of life post-surgery [3]. While much literature is largely focused on preventing devastating outcomes such as endophthalmitis [4,5,6], it is important not to neglect side effects that increase morbidity such as tear disorders producing optical aberrations [7] and meibomian gland dysfunction [8]. DED continues to develop [9] or worsen [10,11] following cataract surgery despite the improvement of cataract surgery techniques. Complete prevention is difficult as several factors influence the development of DED post-cataract surgery. Intra-operative microscopic light and heat [12], administration of Non-Steroidal Anti-Inflammatory Drugs [13], duration of surgery [14], incisional corneal nerve damage [15], repeated intra-operative ocular surface drying and irrigation [16], use of preservatives post-operatively [17], use of aminoglycosides [18], worsening of pre-existing dry eye, and lack of lid hygiene before and after surgery [19] are all associated with increased risk of DED following cataract surgery.

Several studies have shown that post-surgerical DED is transient, with symptoms and parameters worsening within the early post-surgical period and followed by subsequent improvement [20,21]. For example, Choi et al. reported worsening dry eye signs at 1 month and 3 months post-operative compared to baseline [20]. Similarly, Kasetsuwan et al. showed worsening of dry eye signs and symptoms on day 7 post-cataract surgery, with improvements in 30 days and 3 months [21].

However, such studies lack evaluation in the medium and longer term (beyond 3 months) and it was uncertain if dry eye parameters had returned to baseline [10]. It was also unknown if these patients would experience worse dry eye signs and symptoms in the long-term. Hence, we aim to investigate whether a history of cataract surgery amongst patients without known risk factors of DED would significantly worsen tear film-related signs and symptoms within 6 months to 5 years after surgery.

## 2. Materials and Methods

### 2.1. Study Design

This is a cross-sectional study conducted in Singapore National Eye Centre, Singapore, based on prospective clinical audit data of consecutive new patients presenting to a dry eye referral clinic. They presented to the clinic from July 2006 to April 2021. According to the Human Biomedical Research Act in Singapore, data collected for the purpose of clinical care audit are not considered a research study and hence do not require approval by an institutional review board or informed consent. Regardless, the study was conducted in line with Tenets of Declaration of Helsinki for human research. Patients and the public were not involved in the design, conduct, report, or dissemination of plans of research. This study was done in accordance with the STROBE guidelines.

### 2.2. Participants

**Figure 1:**
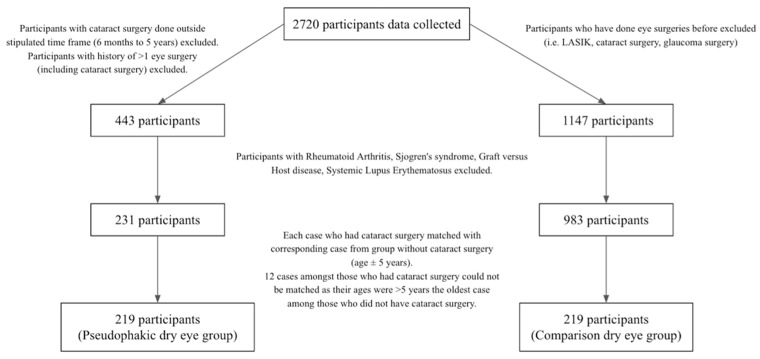
Flowchart of participant recruitment.

In this study, 2720 participants’ data were collected on the first consultation. The study population consisted of those who had cataract surgery within the past 6 months to 5 years in at least one eye. Patients with history of glaucoma surgery, LASIK, Rheumatoid Arthritis, Sjogren’s syndrome, Graft versus Host disease, Systemic Lupus Erythematosus, or incomplete data were excluded (Figure 1).

After excluding the above patients, we matched the age of each pseudophakic dry eye case (*n* = 231) with a corresponding comparison case with no history of cataract surgery (age ± 5 years) and applied the same exclusion criteria to the comparison dry eye group. However, the twelve oldest participants from the cataract group could not be matched because their ages were more than 5 years older than the oldest patient in the comparison group.

Results from a total of 438 eyes were evaluated (219 post-cataract surgery, 219 without eye surgeries). Only one eye in each patient was analysed. In the event cataract surgery was done on both eyes within the defined time frame, the eye with more severe dry eye signs was chosen for analysis. Similarly, among those without eye surgeries (comparison dry eye group), the eye with worse results was evaluated. The duration of ocular discomfort was evaluated with this question: “For how long have you had irritation or discomfort in one or both eyes before this consultation?”. This is compared to the date of cataract surgery for evaluation of the temporal relationship between onset of ocular discomfort and cataract surgery. The date of surgery in participants with bilateral cataract surgery done at different times was defined by the date of surgery done for the study eye. The study eye refers to the eye with more severe dry eye signs, as mentioned above.

### 2.3. Study Outcomes

Outcomes evaluated include symptoms and signs of dry eye and tear film disorder such as Ocular Surface Disease Index (OSDI), Schirmer’s I test score, Tear Break-up Time (TBUT), number of liquid-expressing glands in the lower lid, cornea staining severity, corneal sensation, meibum character, and follicle grade.

#### 2.3.1. Questionnaire

Symptoms of dry eye disease were evaluated using the Ocular Surface Disease Index (OSDI)© questionnaire (Allergan, Inc., Irvine, CA, USA). Briefly, the OSDI questionnaire consisted of 12 questions, each question graded from 0 to 4. For standardisation of responses, grade of 0 was defined to be none of the time, grade of 1 was defined as at least once a month but less than once a week, grade of 2 was defined as at least once a week, grade of 3 was defined as more than once a week but less than once a day and grade of 4 was defined as at least once a day. The total OSDI scores on the scale of 0 to 100 were then calculated with the OSDI© (Allergan, Inc., Irvine, CA, USA) formula: (sum of scores) × 25/(12 questions), with higher scores representing greater severity of symptoms [22].

#### 2.3.2. Tear Break-Up Time

The fluorescein tear breakup time (TBUT) was performed as in previous studies. Briefly, a minimally wet (saline) fluorescein strip applied on the lower fornix was used to instill fluorescein dye and the grading was performed in five corneal zones under slit-lamp microscopy [23,24].

#### 2.3.3. Schirmer’s I Test

The test was performed as in a previous publication. Briefly, the test was done with standard 5 mm wide test strips (Clement Clark^®^, Essex, UK) with a notch for folding without prior anaesthesia. The Schirmer’s strip was folded at the notch and applied over the temporal inferior eyelid. After 5 min, the reading in millimeters (mm) was recorded as the length of wetting of the strip from the notch [8].

#### 2.3.4. Meibomian Gland Dysfunction (MGD) Examination

A standard pressure was applied on the lower lid using a Meibomian Gland Evaluator and the number of glands expressing liquid meibum in the lower eyelid was counted [25].

The character of meibum secreted was evaluated by one ophthalmologist using slit-lamp microscopy, using a firm pressure on the upper eyelid. Character of meibum secreted was graded as follows: 0 = clear meibum, 1 = coloured meibum, 2 = viscous meibum, 3 = unexpressible gland [26].

#### 2.3.5. Fluorescein Corneal Staining

Fluorescein dye was administered in the bulbar conjunctiva using a sterile fluorescein strip. Patient was told to blink several times and the cornea was then observed under the slit lamp microscope using cobalt blue light. Consistent recording of staining severity was facilitated using the Baylor Score [27].

#### 2.3.6. Other Clinical Examination

Subtarsal papillary reaction [28] was examined using a slit lamp biomicroscope. In the upper eyelid, subtarsal conjunctival papillary grading was performed after eversion of the upper eyelid. The tarsal plate was observed under the slit lamp microscope and graded in an ordinal scale from 0 to 3 using the Cornea and Contact Lens Research Unit (CCLRU) grading system [29]. The lower eyelid papillary grading was similarly assessed and graded by pulling down the lower eyelid, rotating it to expose the inferior fornix. Corneal sensitivity was also screened using a cotton wisp [30].

### 2.4. Statistical Analysis

Statistical analysis was performed using StataCorp. 2013 (Stata Statistical Software: Release 13.1. College Station, TX: StataCorp LP). Statistical significance was at two-tailed *p*-value of 0.05. Student’s *t*-test was used to evaluate mean differences of continuous variables such as OSDI score, Schirmer’s I test score, TBUT, number of liquid-expressing glands, and Baylor score. Some continuous variables were categorised into binary categories according to meaningful clinical thresholds and their associations were re-evaluated using Pearson’s Chi-square test via a 2 × 2 table. OSDI score was categorised into ≥33, signifying severe dry eye symptoms, and <33 as the comparison group [26]. To ensure reasonable distribution between the groups compared, frequency of individual OSDI symptoms was categorised into <1 episode a week or ≥1 episodes a week. This may also indicate the severity of OSDI symptoms. Among participants with previous cataract surgery, a proportion of participants with more frequent blurring of vision (≥1 episode a day) was evaluated and compared to the onset of ocular discomfort in relation to cataract surgery. Corneal sensation was divided into two categories: decreased or normal. Schirmer’s score was divided into two categories: ≤8 mm as a measure of aqueous tear deficiency and >8 mm in comparison [31]. TBUT was dichotomised into <5 s, signifying tear instability, and ≥5 s in the comparison group [26]. Papillary grade was dichotomized into two categories—grades 0 to 1 (mild or normal) and grades 2 to 4 (increased papillary or follicular reaction) [29].

Two multiple logistic regression models were performed with Schirmer’s I test grade (defined as ≤8 mm or >8 mm) and blurred vision frequency (defined as <1 episode per week or ≥1 episodes per week) as dependent variables. Covariates of the models were added incrementally.

## 3. Results

The mean post-operative period for pseudophakic group was (2.28 years ± 1.51 years). Tear film parameters associated with DED were not significantly different between the pseudophakic dry eye group and the comparison group (Table 1). Corneal staining (total Baylor scores and Baylor scores in each of the five corneal zones) was not associated with the history of cataract surgery (all *p* > 0.05).

We investigated the effect of cataract surgery on post-operative dry eye symptoms in greater detail. Although the OSDI score was not found to significantly differ between the two groups (Table 1), approximately half of the pseudophakic dry eye group (50%) experienced frequent blurred vision (at least once a week), compared to 38% in the comparison group (*p* = 0.009) (Table 2) when evaluating the individual symptoms in the OSDI Questionnaire. The other symptoms in the OSDI were not different from the comparison group (all *p* > 0.05).

The temporal relationship between dry eye symptoms and cataract surgery among the pseudophakia dry eye group was evaluated against the severity of blurred vision [Table 3]. Out of 219 patients from the pseudophakic dry eye group, 66 patients had ocular discomfort before surgery (mean duration 6.07 years ± 12.9) and 143 had discomfort after the surgery (mean duration 1.20 years ± 1.48). Ten patients could not recall onset of ocular discomfort and were hence removed from further subgroup analysis. Among those with ocular discomfort prior to surgery (*n* = 66), 33% experienced blurred vision at least once a day compared to those without symptoms (*n* = 148) prior to surgery (20%) (*p* = 0.003).

Categorical clinical parameters were evaluated with Pearson’s Chi-square Test [Table 4]. We found that 34% of post-operative participants had a Schirmer’s I test score of >8 mm compared to 25% of the comparison group (*p* = 0.015). This relationship remained significant when a maximum of 25 mm was applied to both groups to exclude those with marked reflex tearing induced by the Schirmer’s strip placement (*p* = 0.021). However, the mean magnitude of the Schirmer’s I test score was not significantly different at 6.5 mm and 7.5 mm [Table 1].

We first performed a logistic regression with blurring grade as a dependent variable [Table 5]. The model included the following covariates: surgery status (pseudophakic dry eye or comparison dry eye group), Schirmer’s grade, gender, and age. A history of cataract surgery was still significantly associated with increased frequency of blurring after adjustment for covariates.

Another logistic regression was performed with Schirmer’s I Test grade as the dependent variable. Similarly, the model included surgery status (post-cataract surgery or comparison dry eye group), blurring grade, gender, and age as independent variables [Table 6]. History of cataract surgery was still significantly associated with increased Schirmer’s I after adjustment.

To examine whether blurring is related to reflex tearing and increased Schirmer’s, a separate analysis showed a weak positive trend for association of increased blurring grade with higher Schirmer’s grade, although not statistically significant (*p* = 0.564).

## 4. Discussion

### 4.1. Summary of Significant Findings

This study has evaluated the effects of cataract surgery on clinical tear film parameters in the medium- to long-term post-surgery and showed that 50% of participants who had undergone cataract surgery experienced more frequent blurred vision (≥1 episode a week) compared to 38% of those without any cataract surgeries (*p*-value = 0.009). Among those with symptomatic dry eyes prior to surgery, 33% experienced blurred vision at least once a day compared to 20% of those without symptomatic dry eyes prior to surgery (*p* = 0.003). The increased frequency of blurring may be partly caused by tear instability or increased reflex tearing, reflected by mildly increased Schirmer’s I test score among the post-operative group, though the increase was not clinically significant.

### 4.2. Comparison with Literature

In an observation study done by Garg et al. in India, OSDI scores and tear film parameters of 120 participants were evaluated pre-operatively and at 1-week and 1-month post-cataract surgery. Like our study, those with ocular or systemic conditions that could contribute to dry eyes were excluded. It was found that patients who underwent cataract surgery had worse dry eye grade according to OSDI scale 1-week post-operation compared to pre-operative grades regardless of type of cataract operation. Improvement was made when measured at 1 month, but OSDI scores did not return to baseline and were still significantly worse than preoperative levels (*p* < 0.001) [32]. In our study, which evaluated a longer period after surgery, similarly, we found that a greater proportion of post-operative patients had more frequent blurred vision than other dry eye patients with no history of eye surgery at 6 months to 5 years after surgery, though we did not find any difference in the overall OSDI. Similarly, Jung et al. also found significant deterioration of OSDI scores after 1 month of cataract surgery, regardless of pre-operative Meibomian Gland Disease (MGD) status. The observational study conducted in South Korea recruited patients with and without MGD prior to cataract surgery and those with ocular or systemic risk factors for dry eyes were excluded. However, no special instructions were given for post-operative lid hygiene or massage, akin to our study where proper lid hygiene was not routinely done. According to Jung et al., patients with more severe MGD prior to surgery had worse ocular surface parameters 1 month after surgery compared to those with mild or no MGD prior to surgery [33]. This correlates with our findings which found that those with ocular discomfort prior to surgery had more frequent blurred vision than those without ocular discomfort prior to surgery, although our study evaluates a longer period after cataract surgery. However, worsening of OSDI scores compared to pre-operative levels has been found to persist up to and beyond 6 months in an observational study conducted by Xue et al. which had similar exclusion criteria compared to our study. Of note, the study also excluded those with severe dry eyes and complicated cataract surgeries [34].

One mechanism explaining increased severity of blurred vision post-cataract surgery includes post-operative development or worsening of existing MGD. MGD may cause intermittent reflex tearing or compensatory hypersecretion of aqueous tears [35]. Jung et al. found that ocular surface parameters and tear cytokine levels worsened after cataract surgery, and the extent of MGD aggravation post-cataract surgery depended on pre-operative MGD status [33]. It is likely that cataract surgery patients had underlying MGD pre-operatively, whether symptomatic or not, since age is a risk factor for both MGD development and cataract disease [36,37]. In our study, there was no significant difference in the mean number of lower lid meibomian glands expressed among the pseudophakic dry eye group (1.80 ± 2.60) compared to the comparison dry eye group (1.70 ± 2.48) (*p* = 0.658). However, pre-operative MGD status was not clinically examined in this study. Furthermore, Han et al. also found that there may be a change in meibomian gland function without structural changes post-cataract surgery [35]. Other mechanisms such as over accommodation, abnormal tear spreading, or blinking may also cause blurring. In addition, the level of inflammation may have increased after the surgery [38], leading to irritation and increased reflex tearing. Increased inflammation may be due to MGD or damage to ocular surface (goblet cells), exposure keratopathy [14], toxicity from povidone iodine preparation [39], or photic damage from microscopic light [12].

Schirmer’s I test score is an objective variable commonly used to assess DED. Li et al. found markedly decreased Schirmer’s I test score at 1 month compared to pre-operative levels, which showed improvement at 3 months, but did not return to baseline pre-operative levels [10]. Similarly, Liu et al. showed a reduction of Schirmer’s I test score 30 days after phacoemulsification [40]. Our result suggests increased basal and reflex tear production among the pseudophakic dry eye group, though the increase of 1 mm in the Schirmer’s I test score was not statistically or clinically significant.

A study reported worsening of corneal epithelial staining grade in the early post-operative period. Li et al. found corneal fluorescein staining peaked 1 month after surgery and mostly returned to baseline at 3 months [10]. Over a longer post-surgery period in our study, staining scores were not different between the pseudophakic and comparison dry eye groups.

### 4.3. Strengths and Weaknesses of Study

Having a large database allows the study of patients in the long-term post-operative period. While the onset of subjective ocular discomfort in relation to cataract surgery was evaluated in this study, the dry eye diagnostic status prior to surgery was not known. The study design also prevented assessment of the progression of dry eye disease in the early, medium, and long-term period after cataract surgery, as well as incident rates of dry eye diagnosis post-surgery. Furthermore, this clinic is a selected population presenting with dry eyes which may not be representative of general patients going for cataract surgery. Data regarding type of cataract surgery were not collected due to potential recall bias. Associations in cross-sectional studies also cannot prove causation.

Another factor that may influence tear film parameters and blurred vision includes lacrimal drainage system obstruction [41]. In the event patients were suspected of lacrimal drainage obstruction, e.g., constant watering or elevated tear meniscus, syringing would have been ordered. This was, however, not encountered in this retrospective study.

### 4.4. Potential Clinical Implications

This study shows that effects of cataract surgery are not limited to the short term. Our findings may have significant implications on patient satisfaction after surgery and patients should be counselled about this undesirable outcome preoperatively, especially if they have symptoms suggestive of dry eyes at pre-operative assessment. Several pre-operative, intra-operative, and post-operative measures should be taken to limit the development of post-operative dry eye disease [42].

## 5. Conclusions

This study shows that cataract surgery did not worsen dry eye signs in the long-term post-surgical period. However, those with a history of cataract surgery within the past 6 months to 5 years experienced increased frequency of blurred vision compared to other dry eye patients with any eye surgeries, especially patients with ocular discomfort suggestive of dry eyes prior to surgery. While it is reassuring that such cases occur without other signs of ocular surface damage, more research should be done to investigate the cause of intermittent blurring.

## Figures and Tables

**Table 1:** Demographics and clinical parameters (continuous variables) in post-cataract surgery and comparison group.

	Pseudophakic Dry Eye Group Mean ± Standard Deviation Median (Min, Max)	Comparison Dry Eye Group Mean ± Standard Deviation Median (Min, Max)	*p*-Value ^†^
Age (years)	69.3 ± 7.96 70 (48, 91)	68.1 ± 4.81 67 (53, 91)	0.055
Ocular Surface Disease Index score	31.8 ± 20.9 27.1 (0, 91.7)	31.4 ± 20.0 28.8 (0, 88.5)	0.838
Schirmer’s I Test (mm)	7.51 ± 7.65 6 (0, 37)	6.51 ± 6.79 5 (0, 35)	0.145
Tear Break-up Score (seconds)	2.45 ± 1.59 2 (0, 11)	2.48 ± 1.92 2 (0, 17)	0.865
Number of liquid-expressing glands in lower lid	1.80 ± 2.60 1 (0, 15)	1.70 ± 2.48 1 (0, 15)	0.658
Baylor Score Total	5.16 ± 6.15 3 (0, 25)	5.63 ± 5.76 4 (0, 21)	0.394
Superior	0.398 ± 1.13 0 (0, 5)	0.361 ± 1.16 0 (0, 4)	0.692
Inferior	1.67 ± 1.67 1 (0, 5)	1.79 ± 1.72 2 (0, 5)	0.458
Nasal	0.91 ± 1.49 0 (0, 5)	1.059 ± 1.49 0 (0, 5)	0.296
Temporal	1.18 ± 1.71 0 (0, 5)	1.31 1 (0, 5)	0.437
Central	0.915 ± 1.55 0 (0, 6)	0.827 ± 1.41 0 (0, 5)	0.530

† Two-tailed Student’s *t*-test.

**Table 2:** Effect of cataract surgery on dry eye symptoms.

Frequency of Symptom in a Week	Pseudophakic Dry Eye Group N (%)	Comparison Dry Eye Group N (%)	*p*-Value ^†^
Light sensitivity			
<1 ≥1	139 80 (36.5%)	143 76 (34.70%)	0.690
Grittiness			
<1 ≥1	115 104 (47.5%)	116 103 (47.0%)	0.924
Burning			
<1 ≥1	157 62 (28.2%)	152 67 (30.6%)	0.600
Blurring			
<1 ≥1	109 110 (50.2%)	136 83 (37.9%)	0.009 *
Vision fluctuation with blinking			
<1 ≥1	148 71 (32.4%)	155 64 (29.2%)	0.469
Vision fluctuation with artificial tears			
<1 ≥1	141 77 (35.3%)	138 81 (37.0%)	0.717
Tearing			
<1 ≥1	149 69 (31.7%)	152 67 (30.6%)	0.811
Pain			
<1 ≥1	194 25 (11.4%)	194 25 (11.4%)	1.00
Fatigue			
<1 ≥1	113 104 (47.9%)	113 106 (48.4%)	0.921
Driving			
<1 ≥1	122 97 (44.3%)	123 96 (43.8%)	0.923
Television			
<1 ≥1	118 101 (46.1%)	120 99 (45.2%)	0.848
Wind			
<1 ≥1	134 85 (38.8%)	135 84 (38.4%)	0.923
Aircon			
<1 ≥1	174 45 (20.5%)	165 54 (24.7%)	0.304

† Pearson’s two-tailed Chi-square test; * *p*-value < 0.05.

**Table 3:** Comparison of severity of blurred vision among those with symptomatic dry eyes prior to surgery and those without.

Frequency of Symptom in a Day	Symptomatic Dry Eyes Prior to Surgery N (%)	No Symptomatic Dry Eyes Prior to Surgery N (%)	*p*-Value ^†^
Blurred vision			
<1 ≥1	44 22 (33.3%)	115 28 (19.6%)	0.003 *

† Pearson’s two-tailed Chi-square test. * *p*-value < 0.05

**Table 4:** Effect of cataract surgery: categorical variables.

	Pseudophakic Dry Eye Group N (%)	Comparison Dry Eye Group N (%)	*p*-Value ^†^
Gender			
Male Female	55 (24.6%) 169 (75.4%)	44 (20.1%) 175 (79.9%)	0.313
Ocular Surface Disease Index (OSDI) score			
<33 ≥33	126 92 (42.2%)	122 97 (44.3%)	0.659
Corneal sensation Normal Decreased or absent	213 4 (1.8%)	213 4 (1.8%)	1.00
Schirmer’s I Test (mm)			
>8 ≤8	77 (34.4%) 141 (65.6%)	54 (24.7%) 165 (75.3%)	0.015 *
Schirmer’s I Test (mm)			
>8 ≤8 (max. 25 mm)	67 141 (67.8%)	47 165 (77.8%)	0.021 *
Tear Break-up Time (seconds)			
≥5 <5	18 200 (91.7%)	17 202 (92.2%)	0.794
Meibum character Liquid Viscous or not expressible	151 61 (28.8%)	160 54 (25.2%)	0.411
Follicle grade Superior			
0 1 2	134 60 (27.9%) 21 (9.77%)	120 73 (34.0%) 22 (10.2%)	0.427
Inferior			
0 1 2	153 41 (19.1%) 21 (9.78%)	146 50 (23.0%) 21 (9.68%)	0.786

† Pearson’s two-tailed Chi-square test; * *p*-value < 0.05.

**Table 5:** Multiple logistic regression with blurring grade as the dependent variable.

Parameters	Model 1 ^†^ Odds Ratio (95% Confidence Interval)	Model 2 ^††^ Odds Ratio (95% Confidence Interval)	Model 3 ^†††^ Odds Ratio (95% Confidence Interval)
Cataract surgery status	1.65 (1.13, 2.42) *	1.69 (1.15, 2.48) *	1.66 (1.13, 2.44) *
Schirmer’s grade		1.21 (0.794, 1.84)	1.18 (0.774, 1.81)
Gender			1.02 (0.650, 1.63)
Age			1.02 (0.983, 1.04)

* *p*-value <0.05; ^†^ Adjusted for cataract surgery status (0 = comparison dry eye group, 1 = pseudophakic dry eye group); ^††^ Adjusted for cataract surgery status and Schirmer’s grade (0 = <8 mm, 1 = ≥8 mm); ^†††^ Adjusted for cataract surgery status, Schirmer’s grade, gender (1 = male, 2 = female) and age.

**Table 6:** Multiple logistic regression with Schirmer’s I result as the dependent variable.

Parameters	Model 1 ^†^ Odds Ratio (95% Confidence Interval)	Model 2 ^††^ Odds Ratio (95% Confidence Interval)	Model 3 ^†††^ Odds Ratio (95% Confidence Interval)
Cataract surgery status	0.625 (0.414, 0.944) *	0.614 (0.405, 0.931) *	0.605 (0.398, 0.921) *
Blurring grade		1.16 (0.764, 1.76)	1.14 (0.744, 1.73)
Gender			1.47 (0.910, 2.37)
Age			1.03 (1.00, 1.06)

* *p*-value <0.05; ^†^ Adjusted for cataract surgery status (0 = comparison dry eye group, 1 = pseudophakic dry eye group); ^††^ Adjusted for cataract surgery status and blurring grade (blurring grade: 0 = never, 1 = <1 x/week, 2 = 1 x/week, 3 = >1 x/week, 4 = ≥1 x/day); ^†††^ Adjusted for cataract surgery status, blurring grade, gender (1 = male, 2 = female), and age.

## Data Availability

The data presented in this study may be available on request from the correspondence author.
